# Supplementary material for: Telomerase RNA structural heterogeneity in living human cells detected by DMS-MaPseq
Source: Nat Commun. 2025 Jan 22;16:925. doi: 10.1038/s41467-025-56149-6 (PMC11754830; doi:10.1038/s41467-025-56149-6)
Supplement: Supplementary file 1 — Supplementary Information [file 41467_2025_56149_MOESM1_ESM.pdf]

Supplementary Information

Telomerase RNA structural heterogeneity in living human cells detected by DMS-MaPseq

Nicholas M. Forino[1#], Jia Zheng Woo[2#], Arthur J. Zaug[3,4], Arcelia Gonzalez Jimenez[5], Eva Edelson[6] Thomas R. Cech[3,4*], Silvi Rouskin[2*], Michael D. Stone[5*]

[1]Department of Molecular, Cell, and Developmental Biology, University of California, Santa Cruz, CA, USA
[2]Department of Microbiology, Harvard Medical School, Boston, MA, USA
[3]Department of Biochemistry, University of Colorado, Boulder, CO, USA
[4]Howard Hughes Medical Institute, University of Colorado, Boulder, CO, USA
[5]Department of Chemistry and Biochemistry, University of California, Santa Cruz, CA, USA
[6]Department of Microbiology and Environmental Toxicology, University of California, Santa Cruz, CA, USA

[#] These authors contributed equally to this work.
[*] Corresponding authors

Supplementary Table 1.	Oligonucleotides used in the study.
Supplementary Figure 1.	Replicate DREEM-deconvoluted DMS profiles of hTR t/PK.
Supplementary Figure 2.	Quantitative evaluation of DMS-modification data concordance with predicted RNA secondary structure models.
Supplementary Figure 3.	Replicate DREEM-deconvoluted DMS profiles of hTR CR4/5.
Supplementary Figure 4.	Unguided RNAstructure predictions of hTR CR4/5.
Supplementary Figure 5.	DREEM-deconvoluted DMS profiles of the CR4/5 domain in BJ Fibroblasts.
Supplementary Figure 6.	Comparison of DREEM-deconvolution results across different cell types.
Supplementary Figure 7.	DREEM-deconvoluted DMS profiles of the CR4/5 domain in HeLa cells overexpressing hTERT.
Supplementary Figure 8.	DREEM-deconvoluted DMS profiles of the CR4/5 domain in overexpressed WT hTR.
Supplementary Figure 9.	Population average DMS reactivity of the hTR t/PK domain within biochemically purified telomerase RNP complexes.

Supplementary Table 1. Oligonucleotides used in the study (listed 5'→3')

hTR RT, UMI, partial Nextera	GTCTCGTGGGCTCGGAGATGTGTATAAGAGACAGNNNNGCATGTGTGAGCCGAGTCCTGGGTGC
hTR second strand, UMI, partial Nextera	TCGTCGGCAGCGTCAGATGTGTATAAGAGACAGNNNNGGGTTGCGGAGGGTGGGCCTG
hTR PK amplicon, partial Nextera	TCGTCGGCAGCGTCAGATGTGTATAAGAGACAGTTCGCCCCTCCCGGGGAC
hTR TWJ amplicon, partial Nextera	GTCTCGTGGGCTCGGAGATGTGTATAAGAGACAGTGCCTCCGGAGAAGCCCCG
Forward hTR cloning (pBS-U1-hTR)	TCTAGAACTAGTGGATCCCCCGGG
Reverse hTR cloning (pBS-U1-hTR)	CGAGGTCGACGGTATCGATAAGCTTG
M2 Megaprimer	GGGTGCCACCGCAGAAGCCCCGGGCCGAC
M3 Megaprimer	GCCACCGCAGAAGCGCCGGGCCGACCGCGGCC
Nextera_Primer_A1	AATGATACGGCGACCACCGAGATCTACACTAGATCGCTCGTCGGCAGCGTCAGATG
Nextera_Primer_A2	AATGATACGGCGACCACCGAGATCTACACCTCTGTATTCGTCGGCAGCGTCAGATG
Nextera_Primer_A3	AATGATACGGCGACCACCGAGATCTACACGATCAGCATCGTCGGCAGCGTCAGATG
Nextera_Primer_A4	AATGATACGGCGACCACCGAGATCTACACACAGTATGTCGTCGGCAGCGTCAGATG
Nextera_Primer_A5	AATGATACGGCGACCACCGAGATCTACACGACTGGAGTCGTCGGCAGCGTCAGATG

Nextera_Primer_A6	AATGATACGGCGACCACCGAGATCTACACACTGCATATCGTCGGCAGCGTCAGATG
Nextera_Primer_A7	AATGATACGGCGACCACCGAGATCTACACTTGCATGCTCGTCGGCAGCGTCAGATG
Nextera_Primer_A8	AATGATACGGCGACCACCGAGATCTACACCGAATCCTTCGTCGGCAGCGTCAGATG
Nextera_Primer_B1	CAAGCAGAAGACGGCATACGAGATTCGCCTTAGTCTCGTGGGCTCGGAGATGTGTAT
Nextera_Primer_B2	CAAGCAGAAGACGGCATACGAGATCTAGTACGGTCTCGTGGGCTCGGAGATGTGTAT
Nextera_Primer_B3	CAAGCAGAAGACGGCATACGAGATTTCTGCCTGTCTCGTGGGCTCGGAGATGTGTAT
Nextera_Primer_B4	CAAGCAGAAGACGGCATACGAGATGCTCAGGAGTCTCGTGGGCTCGGAGATGTGTAT
Nextera_Primer_B5	CAAGCAGAAGACGGCATACGAGATAGGAGTCCGTCTCGTGGGCTCGGAGATGTGTAT
Nextera_Primer_B6	CAAGCAGAAGACGGCATACGAGATCATGCCTAGTCTCGTGGGCTCGGAGATGTGTAT
Nextera_Primer_B7	CAAGCAGAAGACGGCATACGAGATGTAGAGAGGTCTCGTGGGCTCGGAGATGTGTAT
Nextera_Primer_B8	CAAGCAGAAGACGGCATACGAGATCAGCCTCGGTCTCGTGGGCTCGGAGATGTGTAT
Northern Blot Probe hTR 130R-27	CTT TTC CGC CCG CTG AAA GTC AGC GAG
Northern Blot Probe hTR 255R-28	GCC TCC AGG CGG GGT TCG GGG GCT GGG C
Northern Blot Probe U1 101R-21	GGG GAA ATC GCA GGG GTC AGC
Northern Blot Probe U2 153R-22	CGA TGC GTG GAG TGG ACG GAG C
LC pA5	TTAGGGTTAGCGTTAGGG

LC pA5-2	AGGGTTAGCGTTAGGG
LC pA5-3	GGGTTAGCGTTAGGG

Supplementary Figure 1

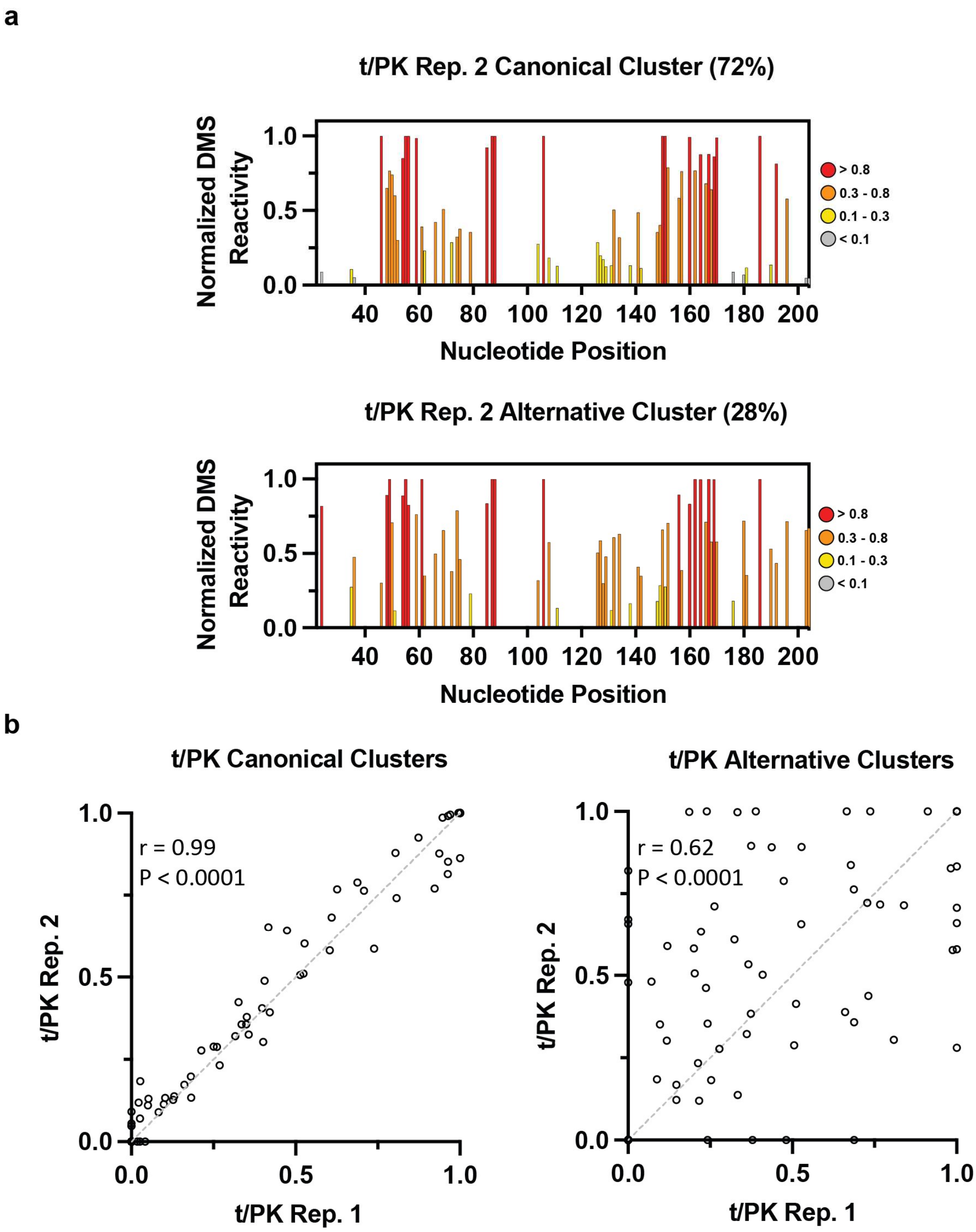

Supplementary Figure 1. Replicate DREEM-deconvoluted DMS profiles of hTR t/PK. **(a)** Normalized DMS reactivity profiles of the clusters predicted by DREEM in a biological replicate experiment. Intensity of DMS reactivity colored according to the provided legend. **(b)** Comparison of DMS reactivities from DREEM-predicted clusters of canonical (left) and alternative (right) conformations of the hTR t/PK domain. Pearson correlation (r) and P values from a two-tailed test are shown. Line of identity is shown as a grey dashed line.

Supplementary Figure 2

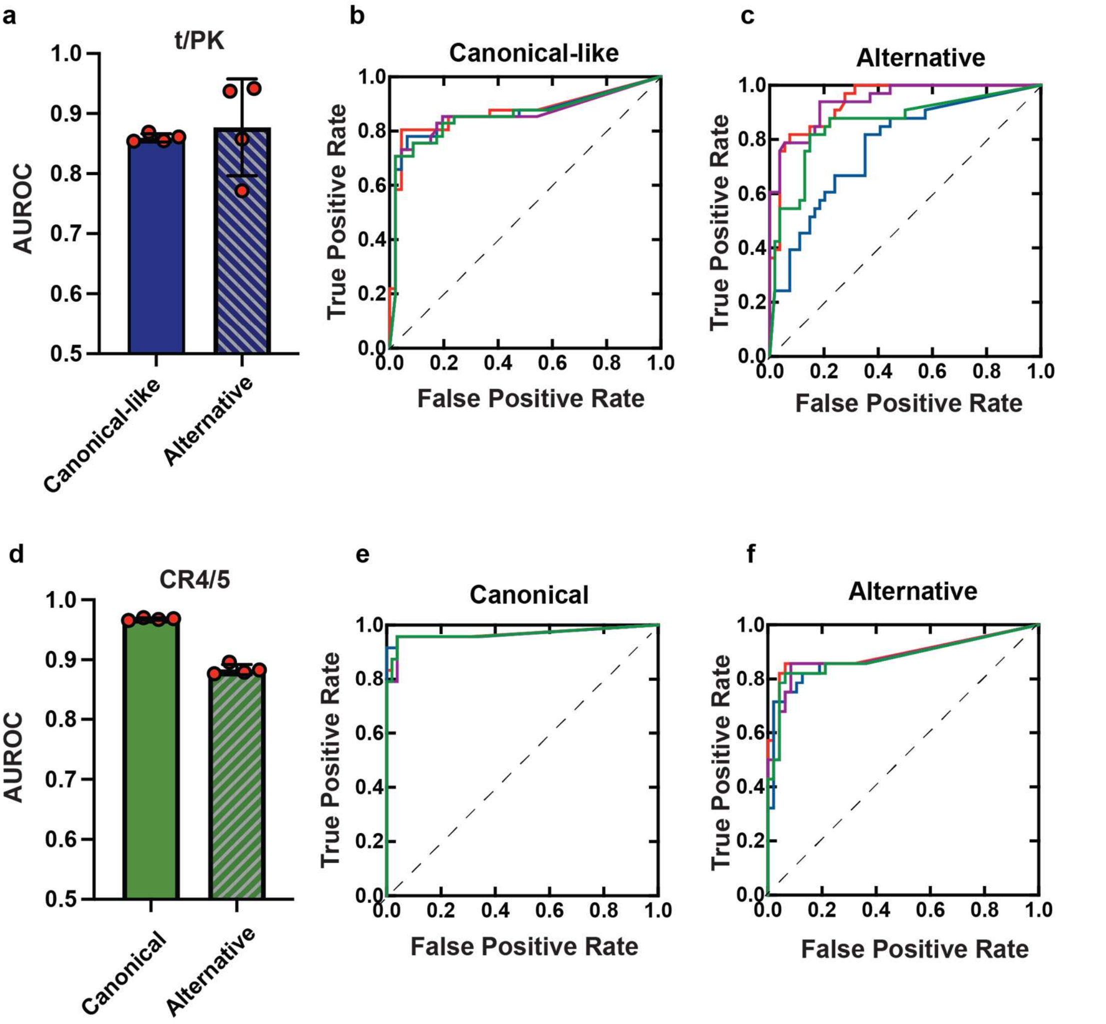

Supplementary Figure 2. Evaluation of DREEM-derived RNA structure models with the area uder the receiver operating characteristic curve (AUROC). (a) Bar plot depicting AUROC values of DREEM-clustered DMS reactivities of the t/PK canonical-like (left, blue bar) and alternative conformations (right, blue hatched bar). Receiver operating characteristic (ROC) curves comparing the DREEM-derived canonical-like t/PK conformation (**b**) and alternative conformation (**c**) with DMS reactivities from their respective DREEM clusters. **(d)** Bar plot depicting AUROC values of DREEM-clustered DMS reactivities of the CR4/5 canonical (left, green bar) and alternative conformations (right, green hatched bar). Receiver operating characteristic (ROC) curves comparing the DREEM-derived canonical CR4/5 conformation (**e**) and alternative conformation (**f**) with DMS reactivities from their respective DREEM clusters.

Supplementary Figure 3

a

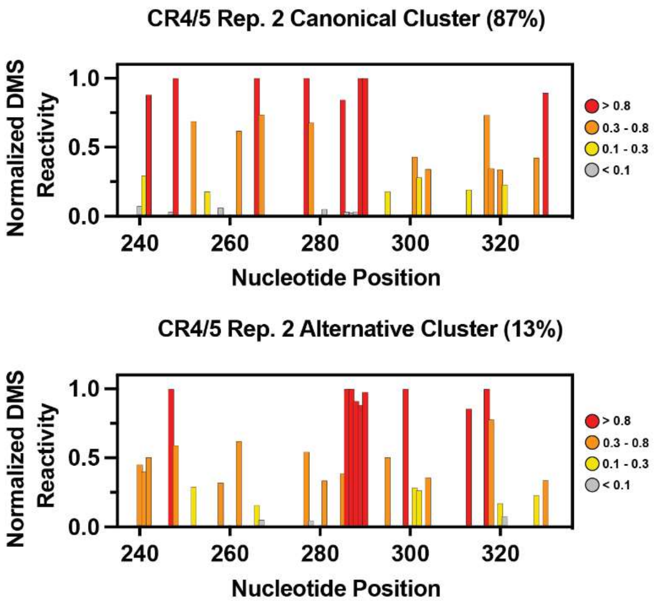

b

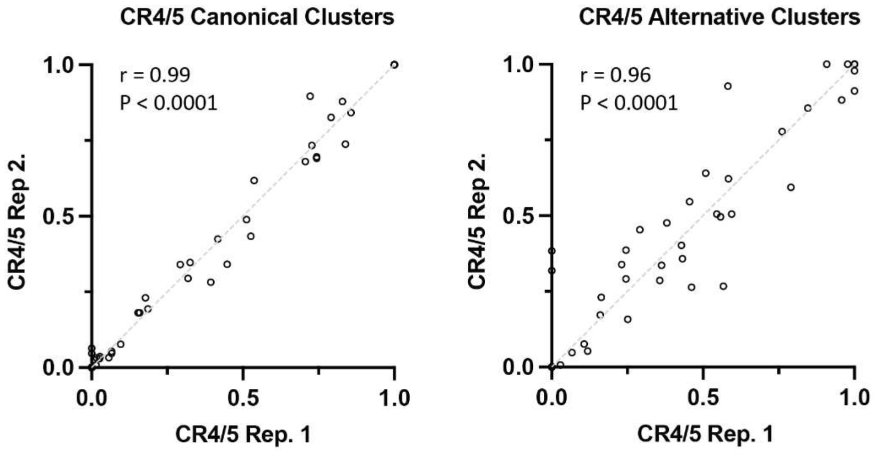

Supplementary Figure 3. Replicate DREEM-deconvoluted DMS profiles of hTR CR4/5. **(a)** Normalized DMS reactivity profiles of the clusters predicted by DREEM in a biological replicate experiment. Intensity of DMS reactivity colored according to the provided legend. **(b)** Comparison of DMS reactivities from DREEM-predicted clusters of canonical (left) and alternative (right) conformations of the hTR CR4/5 domain. Pearson correlation (r) and P values from a two-tailed test are shown. Line of identity is shown as a grey dashed line.

Supplementary Figure 4

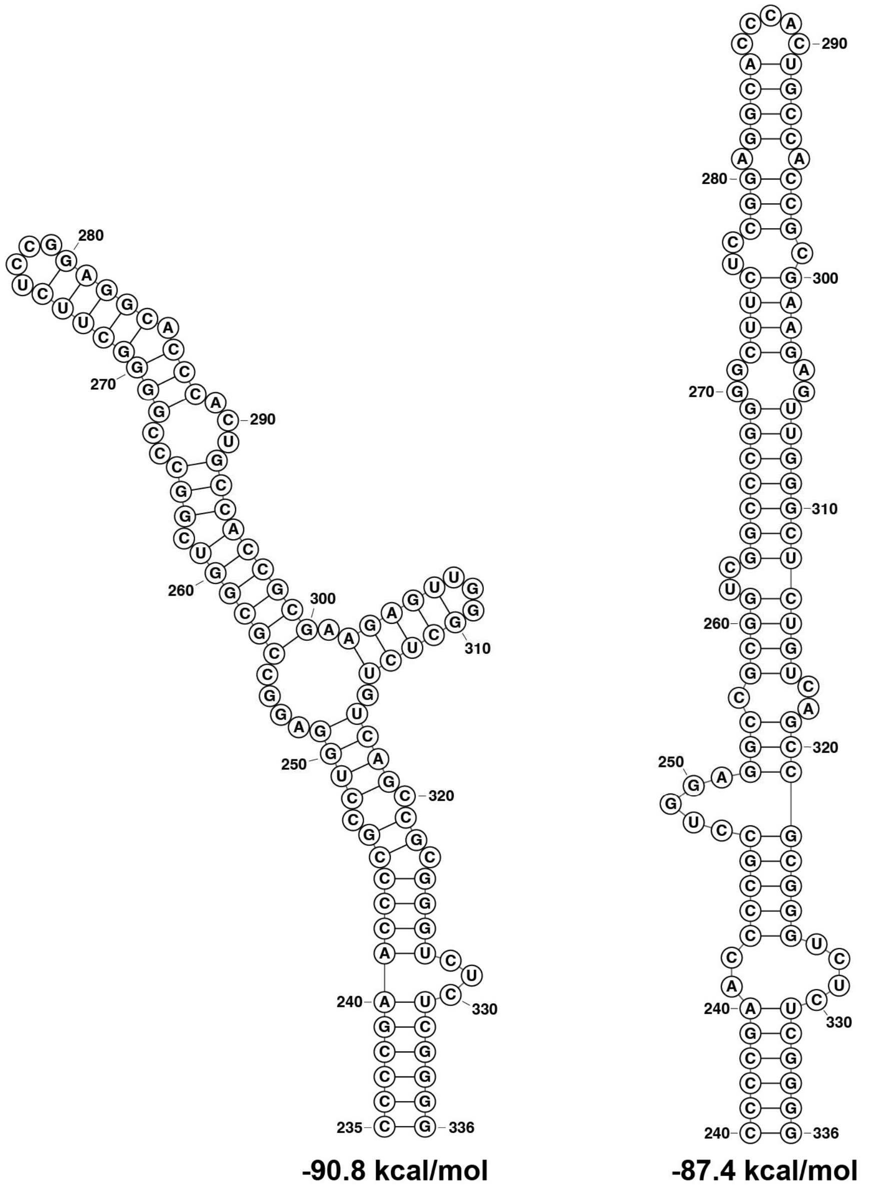

Supplementary Figure 4. Unguided RNAstructure predictions of hTR CR4/5.
Secondary structure predictions of the hTR CR4/5 using RNAstructure default parameters and no DMS reactivity yield the canonical (left) and alternative (right) conformations with similar predicted free energies.

Supplementary Figure 5

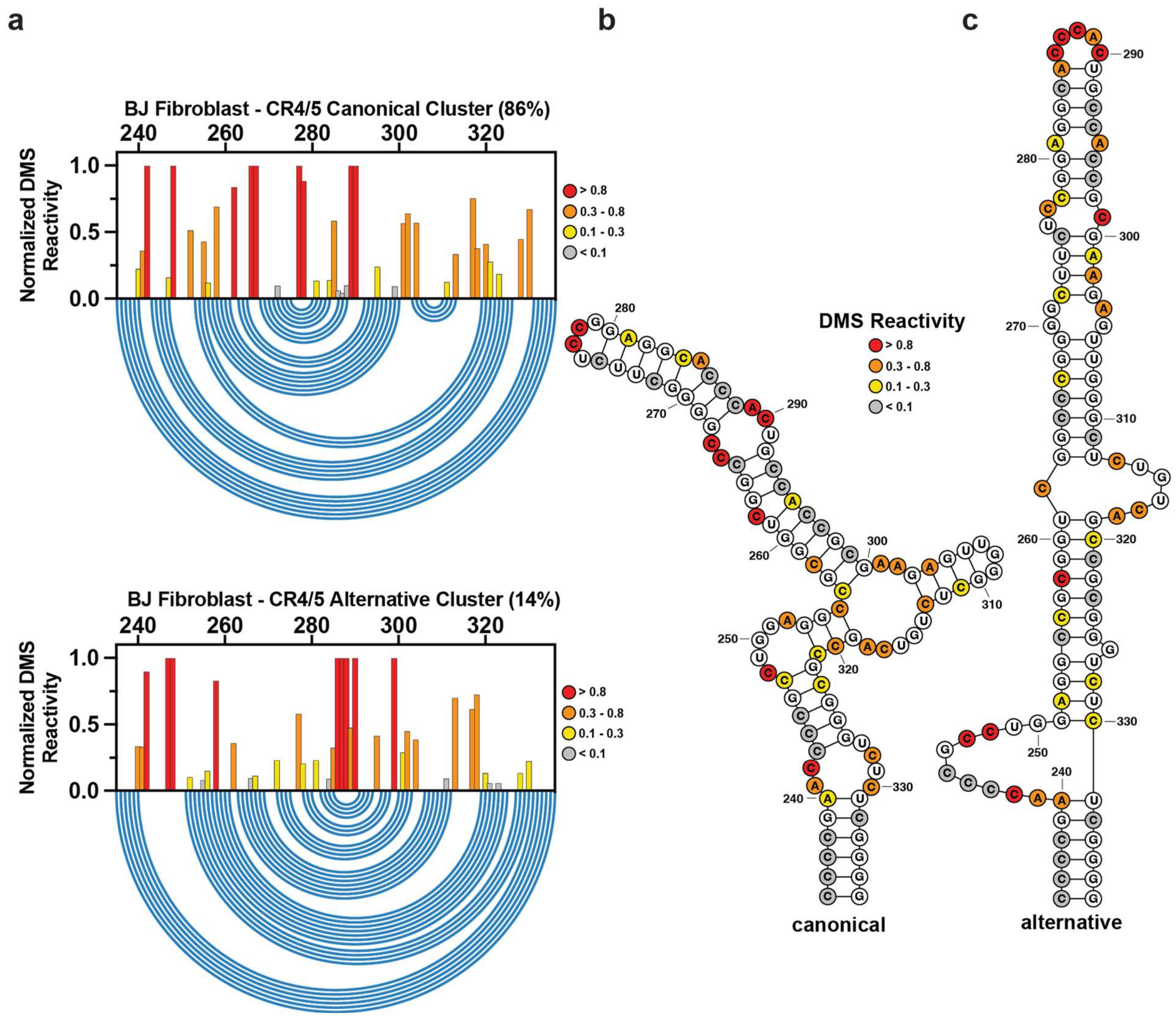

Supplementary Figure 5. DREEM-deconvoluted DMS profiles of the CR4/5 domain in BJ Fibroblasts. (a) Normalized DMS reactivity of the hTR CR4/5 domain in BJ fibroblast cells. Intensity of DMS reactivity according to the provided legend. Blue arcs designate the base pairing pattern of the data-guided predicted CR4/5 secondary structure. **(b)** Data-guided secondary structure prediction of the CR4/5 domain from the canonical cluster of DMS reactivities. **(c)** Data-guided secondary structure of the CR4/5 domain from the alternative cluster of DMS reactivities.

Supplementary Figure 6

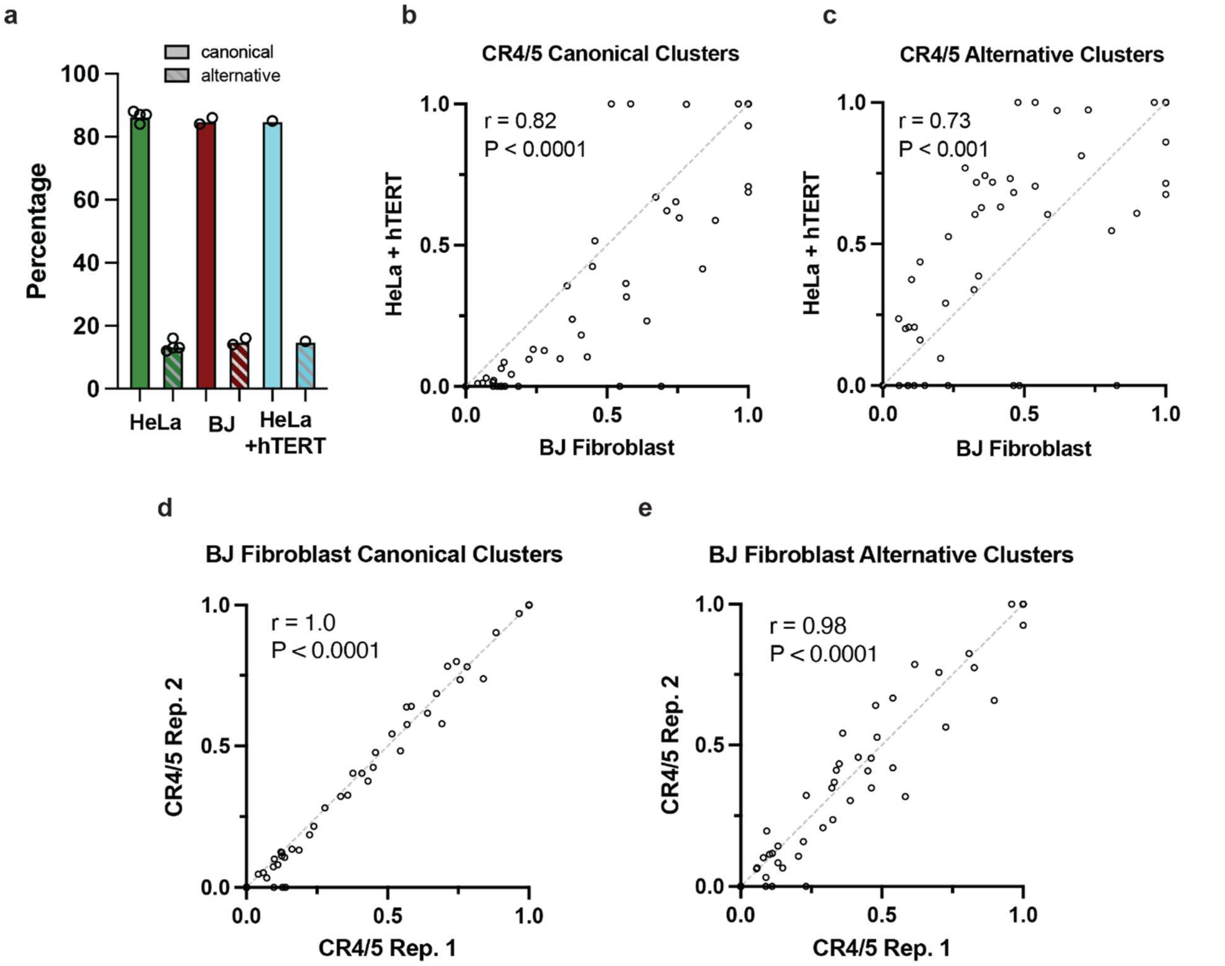

Supplementary Figure 6. Comparison of DREEM-deconvolution results across different cell types. (a) Bar graph depicting the proportion of the canonical conformation (solid bars) and the alternative conformation (hatched bars) of the CR4/5 domain in HeLa cells (green bars), BJ fibroblasts (red bars), and HeLa cells transiently transfected with a plasmid encoding hTERT with an N-terminal FLAG tag (light blue bars). Comparison of DMS reactivities from DREEM-predicted clusters of canonical **(b)** and alternative **(c)** conformations of the hTR CR4/5 domain between HeLa cells overexpressing hTERT (y-axis) and BJ fibroblasts (x-axis). Pearson correlation (r) and P values from a two-tailed test are shown. Line of identity is shown as a grey dashed line. Comparison of DMS reactivities from DREEM-predicted clusters of canonical **(d)** and alternative conformations **(e)** of the hTR CR4/5 domain in BJ fibroblast cells.

Supplementary Figure 7

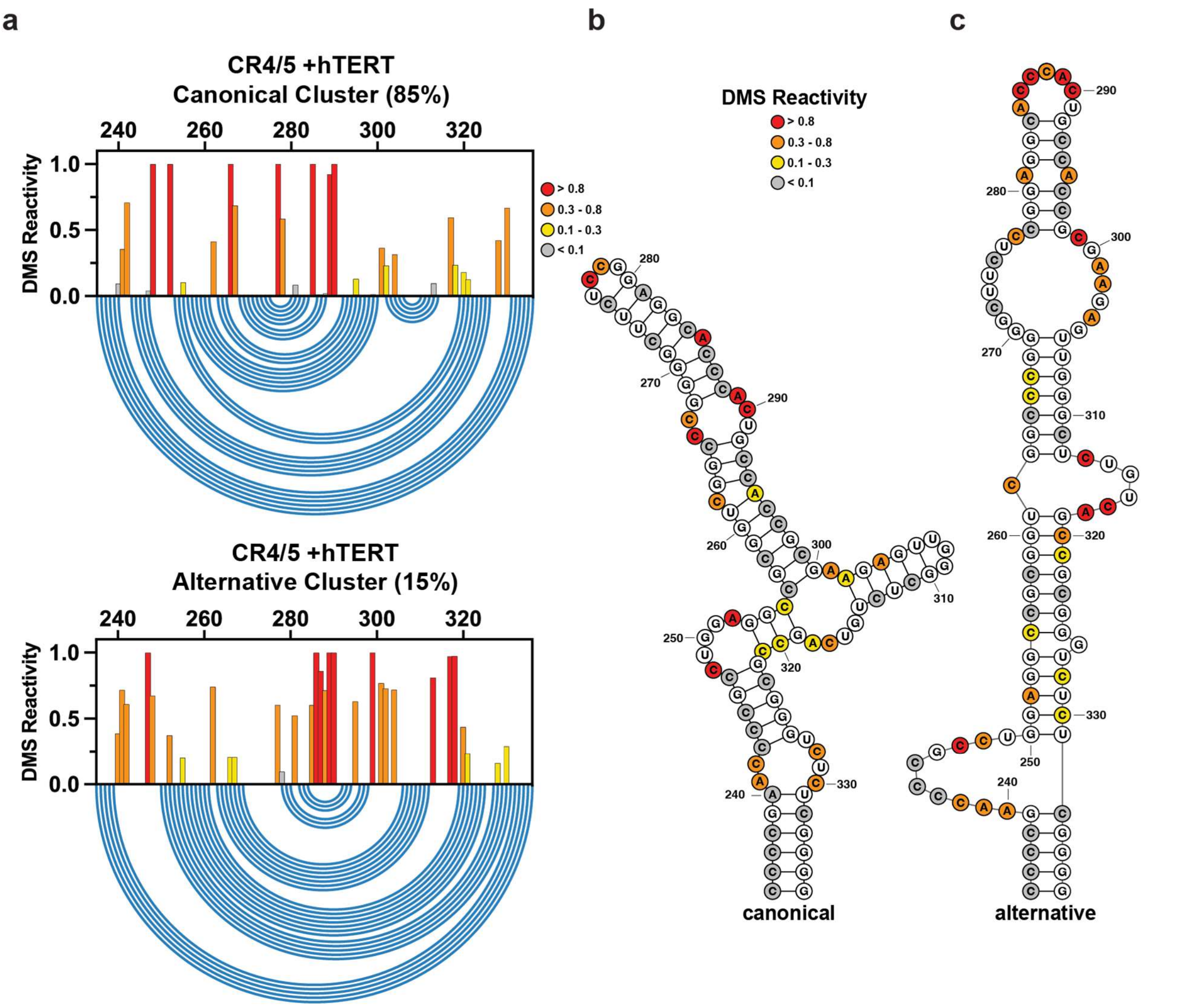

Supplementary Figure 7. DREEM-deconvoluted DMS profiles of the CR4/5 domain in HeLa cells overexpressing hTERT. (a) Normalized DMS reactivity of the hTR CR4/5 domain in HeLa cells transiently transfected with plasmid encoding an N-terminal tagged hTERT. Intensity of DMS reactivity according to the provided legend. Blue arcs designate the base pairing pattern of the data-guided predicted CR4/5 secondary structure. **(b)** Data-guided secondary structure prediction of the CR4/5 domain from the canonical cluster of DMS reactivities. **(c)** Data-guided secondary structure of the CR4/5 domain from the alternative cluster of DMS reactivities.

Supplementary Figure 8

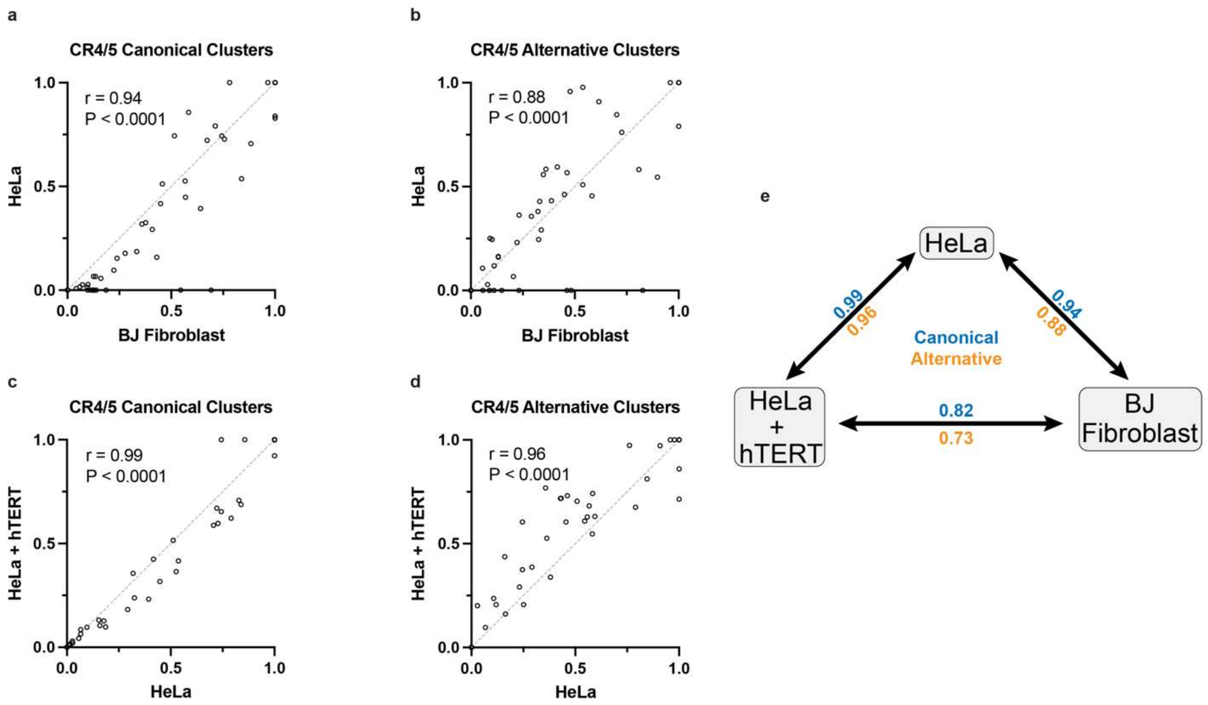

Supplementary Figure 8. Correlation of DMS reactivities from DREEM-predicted clusters in HeLa cells, HeLa cells + hTERT transfection, and BJ fibroblast cells. Comparison of DMS reactivities from DREEM-predicted clusters of canonical **(a)** and alternative **(b)** conformations of the hTR CR4/5 domain between BJ fibroblasts (x-axis) and HeLa cells (y-axis). Comparison of DMS reactivities from DREEM-predicted clusters of canonical **(c)** and alternative **(d)** conformations of the hTR CR4/5 domain between HeLa cells (x-axis) and HeLa cells overexpressing hTERT (y-axis). **(e)** Diagram representing correlation of DMS reactivities of canonical (blue) and alternative (orange) conformations of the hTR CR4/5 domain between HeLa cells, HeLa cells transfected with hTERT, and BJ fibroblasts.

Supplementary Figure 9

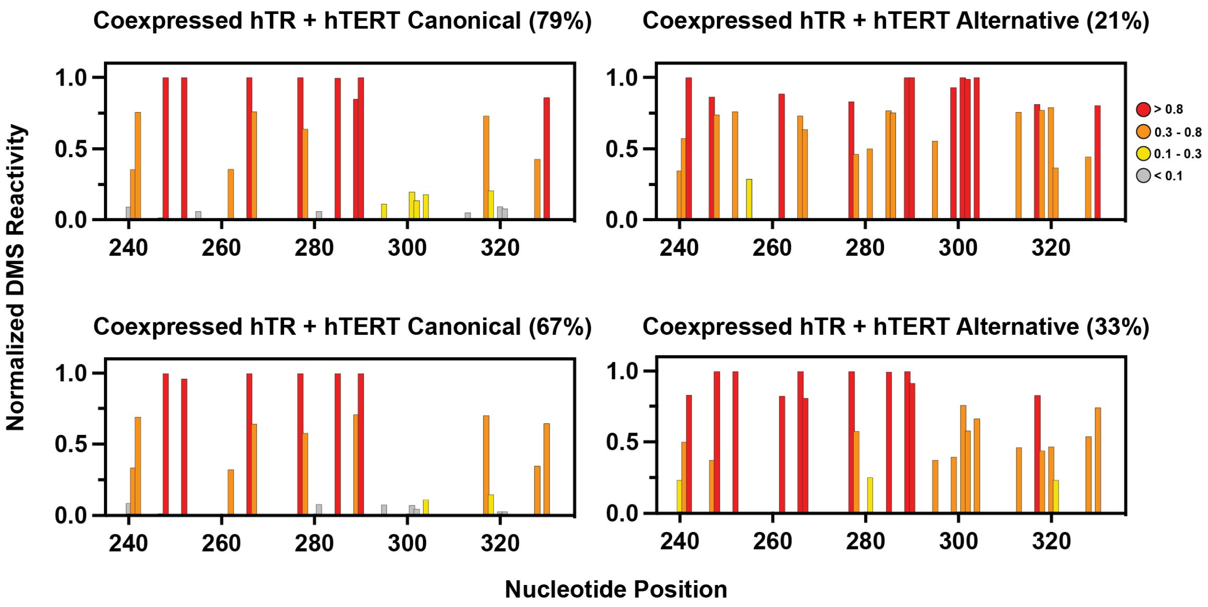

Supplementary Figure 9. DREEM-deconvoluted DMS profiles of the CR4/5 domain in cells co-expressing WT hTR and hTERT. DMS profiles of the two clusters predicted by DREEM from cells overexpressing WT hTR and hTERT. Cluster abundances are depicted as percentages. Results from two independent experiments are shown.

Supplementary Figure 10

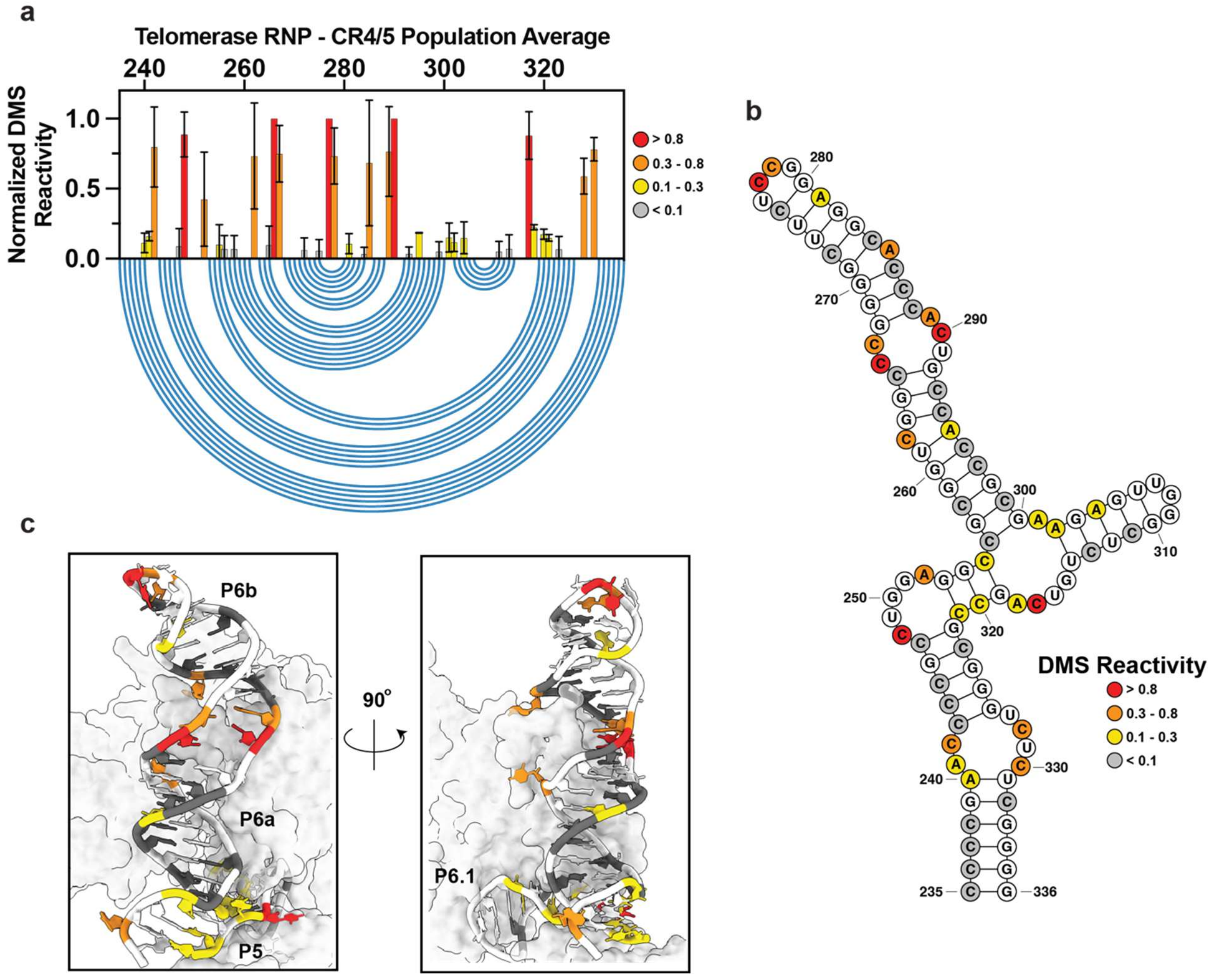

Supplementary Figure 10. Population average DMS reactivity of the hTR CR4/5 domain within biochemically purified telomerase RNP complexes. (**a**) Normalized DMS reactivity of hTR CR4/5 domain at 1% and 2% DMS. Intensity of DMS reactivity colored according to the provided legend. Data are presented as means ± SD. Blue arcs designate the base pairing pattern of the data-guided predicted CR4/5 secondary structure. (**b**) Data-guided secondary structure prediction of the CR4/5 domain from the population average of DMS reactivities. **(c)** DMS reactivity overlaid onto the cryo-EM model of assembled telomerase.
