# Supplementary material for: Telomerase RNA structural heterogeneity in living human cells detected by DMS-MaPseq
Source: Nat Commun. 2025 Jan 22;16:925. doi: 10.1038/s41467-025-56149-6 (PMC11754830; doi:10.1038/s41467-025-56149-6)
Supplement: Supplementary file 2 — Reporting summary [file 41467_2025_56149_MOESM2_ESM.pdf]

nature portfolio

Corresponding author(s): Michael Stone, Silvi Rouskin, Thomas Cech

Last updated by author(s): 12/18/2024

Reporting Summary

Nature Portfolio wishes to improve the reproducibility of the work that we publish. This form provides structure for consistency and transparency in reporting. For further information on Nature Portfolio policies, see our Editorial Policies and the Editorial Policy Checklist.

Statistics

For all statistical analyses, confirm that the following items are present in the figure legend, table legend, main text, or Methods section.

n/a	Confirmed	
☐	☒	The exact sample size (*n*) for each experimental group/condition, given as a discrete number and unit of measurement
☐	☒	A statement on whether measurements were taken from distinct samples or whether the same sample was measured repeatedly
☒	☐	The statistical test(s) used AND whether they are one- or two-sided<br>*Only common tests should be described solely by name; describe more complex techniques in the Methods section.*
☒	☐	A description of all covariates tested
☒	☐	A description of any assumptions or corrections, such as tests of normality and adjustment for multiple comparisons
☐	☒	A full description of the statistical parameters including central tendency (e.g. means) or other basic estimates (e.g. regression coefficient) AND variation (e.g. standard deviation) or associated estimates of uncertainty (e.g. confidence intervals)
☐	☒	For null hypothesis testing, the test statistic (e.g. *F*, *t*, *r*) with confidence intervals, effect sizes, degrees of freedom and *P* value noted<br>*Give P values as exact values whenever suitable.*
☒	☐	For Bayesian analysis, information on the choice of priors and Markov chain Monte Carlo settings
☒	☐	For hierarchical and complex designs, identification of the appropriate level for tests and full reporting of outcomes
☐	☒	Estimates of effect sizes (e.g. Cohen's *d*, Pearson's *r*), indicating how they were calculated

Our web collection on statistics for biologists contains articles on many of the points above.

Software and code

Policy information about availability of computer code

Data collection	No software used.
Data analysis	TrimGalore (V0.6.6, https://github.com/FelixKrueger/TrimGalore) was used to trim reads and filter for a minimum length of 120 nt. Alignment of sequencing reads to the wild-type hTR sequence was performed using Bowtie2 (2.4.4, https://bowtie-bio.sourceforge.net/bowtie2/index.shtml). Conversion of SAM to BAM files were performed using SAMtools (1.16.1, http://www.htslib.org). Ensemble deconvolution was performed with the Detection of RNA Ensembles using Expectation Maximization (DREEM) software (Version 1.0) is available on Code Ocean (https://codeocean.com/capsule/6175523/tree/v1).

For manuscripts utilizing custom algorithms or software that are central to the research but not yet described in published literature, software must be made available to editors and reviewers. We strongly encourage code deposition in a community repository (e.g. GitHub). See the Nature Portfolio guidelines for submitting code & software for further information.

Data

Policy information about availability of data

All manuscripts must include a data availability statement. This statement should provide the following information, where applicable:

- Accession codes, unique identifiers, or web links for publicly available datasets
- A description of any restrictions on data availability
- For clinical datasets or third party data, please ensure that the statement adheres to our policy

The sequencing data, normalized DMS reactivities, and predicted RNA secondary structures generated in this study have been deposited into the NCBI Gene Expression Omnibus (GEO) under accession code GSE245536 (https://www.ncbi.nlm.nih.gov/geo/query/acc.cgi?acc=GSE245536).

Research involving human participants, their data, or biological material

Policy information about studies with human participants or human data. See also policy information about sex, gender (identity/presentation), and sexual orientation and race, ethnicity and racism.

Reporting on sex and gender	NA
Reporting on race, ethnicity, or other socially relevant groupings	*Please specify the socially constructed or socially relevant categorization variable(s) used in your manuscript and explain why they were used. Please note that such variables should not be used as proxies for other socially constructed/relevant variables (for example, race or ethnicity should not be used as a proxy for socioeconomic status).*<br>*Provide clear definitions of the relevant terms used, how they were provided (by the participants/respondents, the researchers, or third parties), and the method(s) used to classify people into the different categories (e.g. self-report, census or administrative data, social media data, etc.)*<br>*Please provide details about how you controlled for confounding variables in your analyses.*
Population characteristics	NA
Recruitment	NA
Ethics oversight	NA

Note that full information on the approval of the study protocol must also be provided in the manuscript.

Field-specific reporting

Please select the one below that is the best fit for your research. If you are not sure, read the appropriate sections before making your selection.

☒ Life sciences ☐ Behavioural & social sciences ☐ Ecological, evolutionary & environmental sciences

For a reference copy of the document with all sections, see nature.com/documents/nr-reporting-summary-flat.pdf

Life sciences study design

All studies must disclose on these points even when the disclosure is negative.

Sample size	We observed good reproducibility of MaPseq data from three different operators using the same experimental and analysis protocols, highlighting the robustness of the MaPseq technique. Experiments were reproduced with at least two independent biological replicates peformed by different scientists. In certain cases, additional replicates were performed as these were the initial observations and therefore experiments were conducted more than twice to ensure reproducibility.
Data exclusions	No data was excluded from this study.
Replication	Experimental protocols such as cell culturing, DMS modification, and library preparation were performed by three different operators using the same experimental protocol. Bioinformatic analysis such as read quality control, read alignment, and DREEM clustering were executed using the same commands and running parameters.
Randomization	Sample size randomization was not necessary to this study as it was performed in a relatively low-throughput manner. Cell culturing was performed in 6-well culture plates, and library preparation was executed in 8-strip tubes, thus we do not expect operational variables such as edge effects to affect sample handling and the downstream data in our study.
Blinding	Due to the exploratory nature of out study, we had no prior assumptions or expectations of the folding behavior of hTR across various cell types and hTR mutation contexts. Thus, we did not blind investigators to the identity of biological samples.

Reporting for specific materials, systems and methods

We require information from authors about some types of materials, experimental systems and methods used in many studies. Here, indicate whether each material, system or method listed is relevant to your study. If you are not sure if a list item applies to your research, read the appropriate section before selecting a response.

Materials & experimental systems

n/a	Involved in the study
☐	☒ Antibodies
☐	☒ Eukaryotic cell lines
☒	☐ Palaeontology and archaeology
☒	☐ Animals and other organisms
☒	☐ Clinical data
☒	☐ Dual use research of concern
☒	☐ Plants

Methods

n/a	Involved in the study
☒	☐ ChIP-seq
☒	☐ Flow cytometry
☒	☐ MRI-based neuroimaging

Antibodies

Antibodies used

Anti-hTERT (600-401-252, Rockland), anti-DKC1 (NBP3-16405, Novus) and anti-GAR1 (NBP2-31742). Secondary antibody was anti-rabbit (711-035-152, Jackson ImmunoResearch, West Grove, PA).

Validation

hTERT antibody 600-401-252 has been evaluated in the following publication as listed on the manufacturer's website: https://www.ncbi.nlm.nih.gov/pmc/articles/PMC10997850/.
DKCI antibody NBP3-16405 produces a single band via Western blot in our study and also in Western blots on the manufacturer's website: https://www.novusbio.com/products/dkc1-antibody-2w2v6_nbp3-16405#datasheet.
GAR1 antibody NBP2-31742 has been used in the following publication as listed on the manufacturer's website: https://www.ncbi.nlm.nih.gov/pmc/articles/PMC6223129/.

Eukaryotic cell lines

Policy information about cell lines and Sex and Gender in Research

Cell line source(s)

ATCC

Authentication

HeLa cells: HeLa- EM2 - 11ht (Cellosourus); received from Cech lab (Boulder)
HEK293T/17 cells: Lot: 70040949. Species determination via short tandem repeat (STR) profiling.
BJ fibroblasts: ATCC CRL2522

Mycoplasma contamination

Cell lines were not tested for mycoplasma contamination.

Commonly misidentified lines
(See ICLAC register)

Name any commonly misidentified cell lines used in the study and provide a rationale for their use.

Plants

Seed stocks

NA

Novel plant genotypes

NA

Authentication

NA
